# Effect of Induced Mechanical Leaf Damage on the Yield and Content of Bioactive Molecules in Leaves and Seeds of Tepary Beans (*Phaseolus acutifolius*)

**DOI:** 10.3390/plants11243538

**Published:** 2022-12-15

**Authors:** Ricardo Cervantes-Jiménez, Marisol Martínez Martínez, Adán Mercado-Luna, Jorge Luis Chávez-Servín, Bárbara Cabello Ruiz, Ángel Félix Vargas-Madriz, Octavio Roldán-Padrón, Mónica Eugenia Figueroa Cabañas, Roberto Augusto Ferriz-Martínez, Teresa García-Gasca

**Affiliations:** 1 Facultad de Ciencias Naturales, Universidad Autónoma de Querétaro, Av. de las Ciencias S/N, Juriquilla 76320, Querétaro, Mexico; 2 Instituto Tecnológico y de Estudios Superiores de Monterrey, Campus Querétaro, Epigmenio González 500, San Pablo 76130, Querétaro, Mexico; 3 Facultad de Ingeniería, Campus Amealco, Universidad Autónoma de Querétaro, Carretera Amealco–Temascalcingo km 1, Col. Amealco 76850, Querétaro, Mexico

**Keywords:** Tepary beans, lectins, protease inhibitors, foliar stress, phenolic compounds

## Abstract

Growing interest has recently been shown in Tepary beans (*Phaseolus acutifolius*) because they contain lectins and protease inhibitors that have been shown to have a specific cytotoxic effect on human cancer cells. Bean lectins offer protection against biotic and abiotic stress factors, so it is possible that mechanical foliar damage may increase lectin production. This study evaluates the effect of mechanical stress (foliar damage) on lectin and protease inhibitor content in Tepary beans. Seed yield was also analyzed, and phenolic content and antioxidant capacity (DPPH and TEAC) were determined in the leaves. An experimental design with random blocks of three treatments (T1: control group, T2: 50% mechanical foliar damage and T3: 80% mechanical foliar damage) was carried out. Mechanical foliar damage increased the amount of lectin binding units (LBUs) fivefold (from 1280 to 6542 LBUs in T3) but did not affect units of enzymatic activity (UEA) against trypsin (from 60.8 to 51 UEA in T3). Results show that controlled mechanical foliar damage could be used to induce overexpression of lectins in the seeds of Tepary beans. Mechanical foliar damage reduced seed production (−14.6%: from 1890 g to 1615 g in T3) and did not significantly increase phenolic compound levels in leaves.

## 1. Introduction

The genus *Phaseolus*, native to the Americas, comprises five cultivated species (*P. vulgaris* L., *P. lunatus* L., *P coccineus* L., *P. polyanthus* Greenm and *P. acutifolius*). *P. acutifolius* is native to Mexico and the southwestern region of the United States. The Tepary bean (*Phaseolus acutifolius*) belongs to the legume family (Fabaceae) and is a species of which human consumption is limited and mostly local [1]. It is a short-cycle annual plant that reaches maturity between 60 and 80 days after germination. It is highly resistant to drought and hot climates, adaptable to high salt concentrations and resistant to many diseases [2,3]. In the last ten years, interest in the Tepary bean has grown due to the type of lectins and protease inhibitors it contains [4].

Lectins are a class of thermolabile proteins of nonimmune origin, binding to specific carbohydrates (reversible). They are cell binders [5,6] and are also involved in plant defense [7]. Lectins have the ability to interact with immune response cells, which gives them immunosuppressive effects. They can also be toxic, since they inhibit the growth of tumor cells and participate in cell adhesion [8]. Due to these characteristics, there has been increasing interest in the properties of the lectins contained in Tepary beans. In various in vitro studies, Tepary bean lectins have shown a specific cytotoxic effect on human cancer cells, with differential cytotoxic activity on various cell lines. Specifically, the findings show that the colon cancer cell lines (CaCo_2_) were the most sensitive, requiring less quantity to reach lethal concentration 50 (LC_50_) and inhibitory concentration 50 (IC_50_), followed by breast cancer cell lines (ZR-75 and MCF-7) [4,9]. In another study, a differential effect was observed between CaCo-2 cells and nontransformed ileum cells (IEC-18) [10]. An inhibitory effect on the proliferation of colon cancer cells (SW-480) has also been reported after 48 h of exposure to Tepary bean lectins [11].

Protease inhibitors, for their part, are essential to the regulation of proteolytic activity and are related in several biological processes of metabolism and cell physiology [12]. In plants, protease inhibitors are indispensable for cellular homeostasis and survival. They play a role in many physiological processes: mobilization of storage proteins; regulation of endogenous enzymatic activities; modulation of apoptosis and programmed cell death; and stabilization of defense proteins or compounds against animals, insects and microorganisms. Various plant proteins, such as protease inhibitors and lectins, are involved in the creation of the protective barrier in the early stages of various types of pathogen infection [13].

Isolating the Tepary bean’s lectin fraction (TBLF) and protease inhibitors requires a long and expensive process to extract, purify and characterize the proteins obtained from the beans, and yields are relatively low (approximately 0.1 g of concentrated lectin fraction from 1000 g of bean flour) [14,15]. Alternatives for increasing the production of the compounds of interest in Tepary beans may be explored, for example, generating stress to the plant. No study has yet been reported that analyzes stress response in the Tepary bean plant, but some studies have analyzed the effect of chewing insects, pests and organisms in other plants. In pine plants, it has been reported that concentrations of nonvolatile resin, volatile monoterpenes and polyphenols in their tissues increased [16] in response to attacks by stem-chewing insects. In addition, the effect of biotic and environmental factors on the phenolic composition and antioxidant activity of *Vitis vinifera* L. has been studied. It was reported that total phenolic content, flavanols, anthocyanins and antioxidant activity in leaves increased with the inoculation of mycorrhizae under high temperature conditions [17]. The effects of different stress conditions on the germination of *Triticum spelta* L. seeds on extractable and bound phenolic compounds and their antioxidant capacity have also been analyzed. Certain combinations of abiotic stress were reported to improve the phenolic profiles and antioxidant activities of germinated seeds [18]. Over time, plants have developed various specific adaptations to adverse conditions, among which are the regulation of a complex cellular and molecular process [19] involving morphological mechanisms, structural characteristics and chemical compounds that are synthesized in such adverse situations. Lectins in particular have been found to be involved in mechanisms that control plant gene expression. This reveals the importance of lectins in plants’ natural defenses [20]. Proteins that have biological functions participate in defense gene combination and duplication, which gives plants an evolutionary advantage by allowing them to create new proteins or synthesize specific proteins more efficiently. Given that the Tepary bean plant uses lectins and protease inhibitors as a protective mechanism against herbivores, it may be of interest to explore whether foliar stress in the form of mechanical damage to the leaves can induce a defense mechanism that increases lectins and protease inhibitors useful in biotechnological applications [21]. The stress that a plant undergoes can influence the physiological adaptation processes it develops, possibly resulting in changes in growth, tissues or cells and modifications in gene expression, including lectins and secondary metabolites [22,23]. The objective of this study was to evaluate the effect of mechanical stress—in the form of foliar damage after flowering and pod filling of Tepary beans—on the content of lectins and protease inhibitors in the seed and the content of secondary metabolites in the leaf. Seed yield, lectins and protease inhibitors were analyzed in the seed, and total phenolic compounds, flavonoids, condensed tannins, anthocyanins and antioxidant capacity (DPPH and TEAC) were analyzed in the leaf.

Our hypothesis is that a certain level of mechanical foliar damage can increase the amount of lectins and protease inhibitors expressed by the plant without significantly affecting seed yield. The results of this study allow us to evaluate the feasibility of increasing the production of lectins in Tepary beans for biotechnological applications through stimuli in the form of mechanical damage to the leaves of the plant after flowering and pod filling.

## 2. Results

The results are presented as follows: first regarding mechanical foliar damage and seed yield, then the content of lectins and protease inhibitors in the seeds and finally the phenolic compounds and the antioxidant capacity present in leaves.

### 2.1. Mechanical Damage and Seed Yield

The average number of total leaves per plant in groups T1, T2 and T3 was observed to be similar, about 60 leaves per plant before applying the treatments (Table 1). The control group T1 was not subject to mechanical damage, while the plants in group T2 received mechanical damage to half the leaves of each plant, with an average of 30 damaged leaves. To group T3, mechanical damage was subjected to 80% of the leaves, with an average of 46 leaves per plant mechanically damaged (Table 1).

The harvest was carried out when the plants were observed to reach an average ratio of 80% dry pods and 20% fresh pods per plant. In all three treatments (T1, T2 and T3), no significant differences (*p* > 0.05) were observed in the content of dry pods during harvest, with about 29 dry pods in each of the three groups (Table 1). In contrast, as the proportion of mechanical damage increased, the number of fresh pods decreased: in T2, fresh pods decreased by 24.10% on average (to 8.5 pods), and in T3, where mechanical damage was inflicted on 80% of the leaves, a decrease of 46.42% (to 6 pods) was observed, in both cases compared to the control group, which showed an average of 11 fresh pods per plant (Table 1). When the total yield of each group was analyzed, a total of 1285 fresh pods were observed in group T1, 1015 fresh pods in group T2 and 717 fresh pods in group T3. Meanwhile, in terms of total dry pods per group, yields were observed to be similar in the T1 and T2 groups (more than 3463 pods), and in the T3 group, 3250 dry pods were counted (Table 2). Tepary bean seed yield decreased when the plant was subject to mechanical foliar damage. In the control group (T1) a production of 1890 g of seed was observed. In the group with mechanical damage to 50% of the leaves (T2), a decrease in production of 12.3% (1658 g of seed) was observed, and in the group with mechanical damage to 80% of the leaves (T3), a decrease of 14.6% (1615 g of seed) was observed (Table 2).

### 2.2. Lectins and Protease Inhibitors

**Figure 1:**
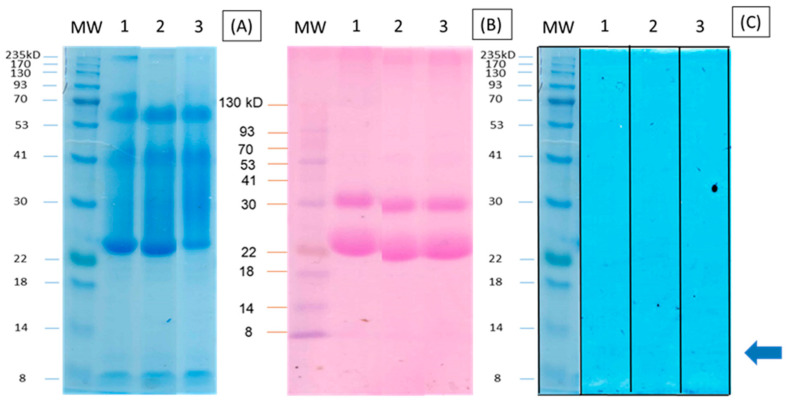
Physicochemical characterization of the LIP-70 extract from Tepary bean seeds. The layout of the lanes is as follows: (1) LIP-70 protein extract from treatment T1; (2) LIP-70 protein extract from treatment T2 and (3) LIP-70 protein extract from treatment T3. Foliar mechanical damage treatments: T1 (0%), T2 (50%) and T3 (80%). (**A**) The electrophoretic profiles of the fractions obtained by SDS-PAGE, in 10% polyacrylamide gels by the Laemmli method, Coumassie stain. (**B**) Identification of glycoproteins present in the polyacrylamide gels following the Periodic-Acid Schiff staining technique (PASS). (**C**) Inhibition zymogram stained with Coumassie blue to observe the presence of trypsin inhibitors.

The analyses performed on seeds to determine the presence of proteins such as lectins and protease inhibitors are shown in Figure 1. The analysis of glycoproteins and banding profile by SDS-PAGE is consistent with the presence of lectins at 30 kDa and the protease inhibitor at 7 kDa. The presence of glycoproteins is also shown.

The lectin binding units (LBUs) of the LIP-70 fraction obtained are shown for each treatment in Table 3. The seeds of the Tepary bean from T2 (50% mechanical foliar damage) presented twice the number of lectin binding units (2560 LBUs), and the T3 group (80% of mechanical foliar damage), five times the number, with values of 6542 LBUs. It was observed that the higher the mechanical foliar damage, the higher the LBU content (Table 3). In contrast, no significant difference was observed in any of the three treatments in terms of units of enzymatic activity (UEA) against trypsin. In the control group, a value of 60.8 UEA against trypsin from the LIP-70 fraction was observed. In the T2 and T3 groups, the observed values were 60.6 and 51.0 UEA, respectively (Table 3). The inhibitors present in LIP-70 are shown in the inhibition zymogram (Figure 1).

### 2.3. Phenolic Compounds and Antioxidant Capacity in Leaves

The content of total phenolic compounds in leaves did not increase in plants subject to mechanical foliar damage. In fact, compared to the average content in the control group (T1), which was 1.374 mg GAE/100 g DM, they were 19.7% lower in the T2 group and 6.5% lower in the T3 group (T1) (Table 4). Similarly, in the group without mechanical damage (T1), a total flavonoid content of 797 mg CE/100 g DM was observed, while in the T2 group, it was 28% lower, and in the T3 group, 4% lower. In other words, mechanical damage to 50% of the leaves caused a significant decrease of 28% (*p* < 0.05) in flavonoid content (compared to the control group T1), while damage to 80 % of the leaves (T3) resulted in a recovery of flavonoid content (764 mg CE/100 g DM) to levels similar to those of plants without mechanical damage. As for the condensed tannin content, readings were similar for all three groups (*p* > 0.05), with values between 1.48 and 1.51 mg CE/100g DM (Table 4). Regarding total anthocyanins, no significant difference was observed between treatments T1 and T2, although in T3 a decrease of almost 8% was observed with respect to the control group, with a value of 0.73 mg C3GE/g DM (Table 4).

**Figure 2:**
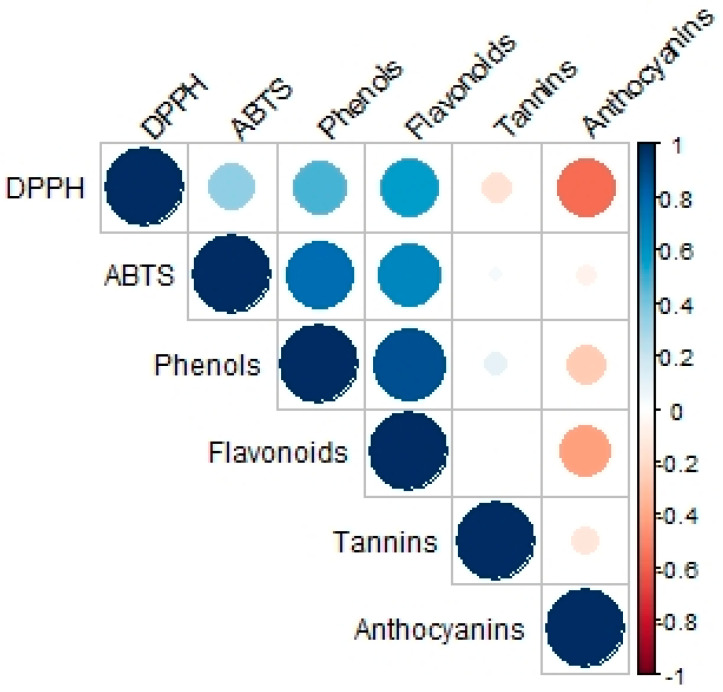
Analysis of Pearson correlation between the content of the components analyzed in the Tepary bean leaf (*Phaseolus acutifolius*). The compounds analyzed in the correlation are 2,2-diphenyl-1-picrylhydrazil (DPPH), (2,2′-azino-bis(3-ethylbenzothiazoline-6-sulfonic acid) (ABTS), total phenols, tannins and anthocyanins. Blue color indicates a correlation close to 1; red color indicates a correlation close to −1. The size of the circle and the intensity of color indicates the correlation between the two variables; the color gradient from blue to red indicates the extent to which they are proportional or inversely proportional to each other, respectively. All tests were performed in triplicate.

A reduction of antioxidant capacity in Tepary bean leaves, evaluated by DPPH and ABTS, was observed in plants subject to mechanical foliar damage. In the DPPH assay, mechanical foliar damage to 50% of the leaves (T2 group) was shown to result in a reduction of 3.7% in antioxidant capacity, and when 80% of the leaves were subject to mechanical damage (T3), a reduction of 5.6% was observed. In the ABTS assay, a reduction in the antioxidant capacity in the leaves of 9.3% was observed in the T2 treatment group, and a reduction of almost 5%, in the T3 treatment group (Table 5). This is consistent with the content of phenolic compounds found in the leaves. The ABTS essay showed a significant positive correlation with phenols and flavonoids, while tannins and anthocyanins did not exhibit significant correlations under ABTS. In the case of DPPH, positive correlations of lesser magnitude were observed between phenols and flavonoids, and negative correlations were observed between tannins and anthocyanins. This indicates that changes in plant composition affect their antioxidant capacity (Figure 2).

## 3. Discussion

In order to minimize the impact on seed yield in production of the Tepary bean, the mechanical foliar damage was carried out when the plant exhibited the following: (a) 100% of its vegetative foliage and 80% of flowering and (b) 100% pod filling with approximately 60% grain maturation. To ensure optimum quality and performance in the products synthesized by the plant, it is important to analyze the moment at which the stress (treatment) should occur [24]. It has been reported that when subject to stress, a plant deploys adaptive response mechanisms that influence its physiological processes, such as slower growth or a change in the amount of photoassimilates or in plant performance [22,23]. One reason plants may have developed these strategies is the wide variety of insects that feed on plant structures [25]. Mechanical damage to 80% of the total leaves of a plant can cause excessive stress on the plant, resulting in the loss of the fruit or inducing its death [26], although better products can be obtained by including some fertilizers and hormones [27]. It was observed that mechanical foliar damage reduced the seed yield of the plant: the T2 treatment group had 232 g less seed weight, and the T3 treatment group had 275 g less than the control group (T1). A study of the effect of early removal on *Vitis vinifera* L. (Tempranillo grape) leaves evaluated phenolic composition, color and morphology in grapes at different stages of development. The authors reported a lower phenolic concentration and smaller seeds in grapes with early leaf removal than in grapes from non-defoliated strains [28]. Similarly, in the present study, a decrease in seed production was observed, along with a reduction in the content of phenolic compounds in leaves and in antioxidant capacity, with mechanical foliar damage to 80% and 50% of the leaves of the plant. Damage to leaves is known to reduce the active photosynthetic surface of plants, producing stress that can affect seed production and nutritional variations [29]. It was observed that when 50% of the leaves of the plant were mechanically damaged, flavonoid content was reduced by 28%, and in turn, antioxidant capacity evaluated by DPPH was reduced by 4%. However, when the mechanical damage was increased to 80% of the leaves of the plant, the stress produced in the leaves was observed to cause a recovery in flavonoid content (767 mg EC/100 g DM) to levels similar to that of the plants without mechanical foliar damage (797 mg EC/100 g DM), along with a 5% increase in antioxidant capacity evaluated by DPPH (263 mg CE/100 g DM). This shows that the synthesis of flavonoids by the leaves may be a stress response to mechanical damage, which allowed the plant to adapt and survive. The rest of the secondary metabolites presented a reduced concentration in plants subject to one of the two treatment methods. As the degree of mechanical damage increased, seed production and the number of fresh pods declined, showing the plants’ adaptation for the purpose of survival, where the ultimate goal is to produce viable seeds.

Metabolites such as phenolic compounds can be altered by minor changes in the ecosystem climate, which can lead to an increase or decrease in certain components of both leaf and seed, in addition to the differences in phenolic compounds that exist between species [30]. Rochín et al. [31] determined the differences in the content of phytochemicals (anthocyanins, flavonoids, total phenolics and tannins), as well as antioxidant capacity, between nine bean species, determining that genetics and climate significantly influence the concentration of these compounds in the seed [25]. In this study, the content of total phenolic compounds and total flavonoids did not behave in a linear fashion: some of the metabolites analyzed in the group subject to mechanical foliar damage to 50% of the leaves declined, and some of them increased in the group subject to mechanical foliar damage to 80%, although without reaching the values of the control group. This may be because the plants studied were in the pod-filling stage, a key moment for the survival of the plant. In this state, the leaf tends to present a certain degree of senescence and the metabolism of the plant is more focused on the seed than on the leaf.

There are no studies in the literature that evaluate the effects of mechanical foliar damage in the Tepary bean plant. There are studies that analyze the effect of chewing insects, pests and organisms that attack other plants. A review of some of these studies follows. In a study in two species of pine *(Pinus pinaster y Pinus radiata*) the effect of two herbivorous chewing insects, the weevil *Hylobius abietis* and the folivore *Thaumetopea pitycampa*, was evaluated [16]. The authors examined the constitutive and herbivore-induced concentrations of total polyphenols, volatile and non-volatile resins, as well as the profile of mono and sesquiterpenes, in the plant stem. They reported that chewing of the stem by the weevil increased concentrations of non-volatile resin, volatile monoterpenes, and polyphenols in stem tissues. They also reported that the folivory by caterpillars did not have significant effects on the defensive chemistry of the needle, but it did prompt a strong increase in the concentration of polyphenols in the stem. In our study, mechanical foliar damage to the leaves was used to approximate the attack of herbivores on the plant, as has been done in other studies [32]. However, the response mechanism may be different in the absence of a predator that may interact with the plant through its secretions and microorganisms present in it [33,34]. In another study, the effect of biotic (mycorrhizal inoculation) and environmental (temperatures: 24/14 °C and 28/18 °C [day/night]) factors on the phenolic composition and antioxidant activity of *Vitis vinífera* L. (Tempranillo grape) leaf extracts was evaluated using grapevines grown in containers in a greenhouse [17]. This work group reports that total phenolic content, antioxidant compounds such as flavanols and anthocyanins, and antioxidant activity of the leaves, all improved with the inoculation of mycorrhizae under high-temperature conditions. They also found that the effects were strongly dependent on the accession tested, which indicated a significant intravarietal diversity in the plants’ response to biotic and environmental factors.

Another study investigated the effects of different stress conditions during germination of spelt seeds (*Triticum spelta* L.) on the antioxidant characteristics of their extractable and bound phenolic compounds. The authors used different types of stress—water volume, salinity, osmolarity, temperatures (10, 20, 30 and 40 °C), and mechanical damage (sandpaper, drawing pins, cutting 1/3 of sprouts and shaking)—during germination [18]. They found that certain combinations of abiotic stresses enhance the phenolic profiles and the extractable and bound antioxidant activities of sprouted seeds. In our study, only mechanical foliar damage was used as an independent variable and other variables such as temperature or the use of foliar elicitors were not evaluated.

The treatments by mechanical foliar damage to the leaves of *Phaseolus acutifolius* correlated with a decrease in weight and size of the seeds and unfavorable changes in the content of total phenolic compounds, total flavonoids, and condensed tannins in Tepary bean leaves, although the values of CPE and defatted raw bean flour (P40 and P70) were similar between treatments and the control group. The T3 treatment group showed significant increases—up to five times—in lectin binding units, which could relate to an expression of lectin in response to mechanical stress, but did not appear to affect expression of trypsin inhibitors. Lectins and protease inhibitors have been reported to be two of the main tools for protection against insects, parasites or microorganisms and an important part of a plant’s overall defensive strategy against predation [35]. An increase in the expression of such proteins may be crucial to the plant’s survival. In the case of Tepary bean, the lectins in the seeds could be a more relevant part of the defense mechanism than the inhibitor proteases, given that the former are present in greater concentrations than the latter [4]. Other studies have reported that pathogen-secreted proteases activate the plant’s immune pathway. It is now known that plant serine protease inhibitors are capable of inhibiting the extracellular serine proteases produced by phytopathogenic microorganisms. For the Tepary bean, the plant may have to interact with the pest insect proteases in order to induce any observable change in the expression of protease inhibitors.

Only lectins were significantly increased in the treatment groups, as reflected in a higher LBU associated with the expression of these proteins. This could be because, in its effort to acclimate to the mechanical foliar damage, the plant underwent a morphological and physiological adjustment to compensate for the stress generated, with a response scale of days to weeks, activating defensive mechanisms that modify cell metabolism to adapt to the new conditions [22]. To achieve a more pronounced increase in the production of protease inhibitors, it would probably be necessary to promote an evolutionary response in the plant. That is, on a much greater time scale than acclimatization and involving several generations of plants.

## 4. Materials and Methods

### 4.1. Reagents and Instruments

Ethanol, methanol, carbonate, aluminum trichloride, hydroxide, sodium nitrate, potassium persulfate, hydrochloric acid, gallic acid, catechin, 6-hydroxy-2,5,7,8-tetramethylchroman-2-carboxylic acid (Trolox), 2,2-diphenyl-1-picrylhydrazyl radical (DPPH), 2,2′-Azino-bis(3-ethylbenzothiazoline-6-sulfonicacid) diammonium salt (ABTS) and Folin-Ciocalteu reagent were purchased from Sigma-Aldrich (St. Louis, MO, USA). For colorimetric determinations, a Spectramax 250 Microplate Reader (Molecular Devices, Marshall Scientifica, Sunnyvale, CA, USA) was used.

### 4.2. Location and Soil Preparation

The cultivation was carried out at the Engineering School of the Autonomous University of Querétaro, on the Concá campus, Arroyo Seco, located at 21°26′05.7″ north latitude and 99°38′22.0″ west longitude, at an altitude of 520 m above sea level, and approximately 240 km from the City of Querétaro, Mexico. The soil of the experimental site is sandy loam, with a high content of organic matter. In a delimited area of 500 m^2^ in the open field, subsoil and fallow work was carried out with agricultural machinery. Subsequently, 25-m-long furrows were made 80 cm apart. Mineralized worm humus was used as background fertilizer. A conventional drip irrigation system was placed using a strip of integrated drippers spaced every 20 cm. At the beginning of the cultivation (12–15 days) irrigation was carried out twice a week with a 16 mm diameter strip, with a flow rate of 0.7 L per hour. In total, watering took place 2 h per week (1.4 L per plant per week). Later, in the flowering stage (27 to 45 days), irrigation was increased to 2.8 L per plant per week. Finally, in the last two stages; from flowering to the appearance of the green pod (7 to 15 days), and from flowering to harvesting the seed, irrigation was 2.1 L per plant per week.

### 4.3. Crop Management and Source of Biological Material

**Figure 3:**
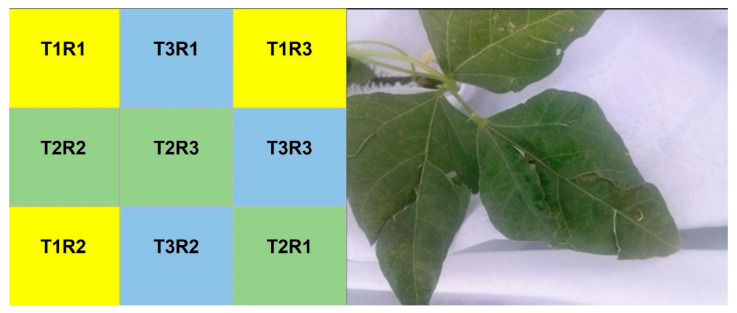
Distribution of experimental blocks and mechanical leaf damage. The image on the (**left**) side shows the distribution of the randomized experimental blocks with 3 replicates. T1 = control group, T2 = mechanical damage was inflicted on 50% of the leaves and T3 = mechanical damage was inflicted on 80% of the leaves; R1, R2 and R3 are the repetitions of each treatment. The image on the (**right**) side shows how the mechanical damage was inflicted, by manually breaking a third of the Tepary bean leaf blade in the midrib area, without separating the petiole from the stem.

The Tepary bean (*Phaseolus acutifolius*) seeds were obtained from the Arizpe community in Hermosillo Sonora, Mexico (30°19′51.96″ N, 110°10′04.3″ O). Sowing was carried out manually (3 July) selecting the white-yellow seeds, discarding seeds that had necrotic tissues or were damaged or incomplete. A seed was placed next to each of the droppers on the strip, 20 cm separating one plant and another in the same row. The flowering of the crop was observed at the beginning of August and the formation of the first pods was observed from the second week of August. An experimental design with random blocks of 3 treatments (T1, T2 and T3) and three repetitions was carried out. The 3 groups were subject to the same irrigation, light and shade conditions. Each treatment was applied to 120 plants in total, for a total of 360 plants, distributed as follows: T1 was the control group, and no mechanical damage was performed; in T2 mechanical damage was inflicted on 50% of the leaves; and in T3 mechanical damage was inflicted on 80% of the leaves. A plastic headband bearing an identification code was placed at the base of each plant. In order to adequately control the treatments applied, the total number of leaves per plant was counted at different phenological moments to determine the number of leaves in each plant to which the mechanical damage would be applied. The mechanical foliar damage (12 September) consisted of manually breaking a third of the Tepary bean leaf blade on the midrib, without detaching the petiole from the main stem of the plant (Figure 3). This was performed when the plant was in the pod filling stage within the reproductive phase with the following characteristics: 80% flowering, 100% pod filling, 60% grain maturation and 100% of the vegetative foliage. The harvest was carried out when the Tepary bean plant was at 80% of its maturation stage (27 September): 80% of the pods dry, yellow or having pigmented coloration and 20% of pods fresh (at 60% of grain maturation).

The pods were collected in perforated plastic bags to avoid moisture accumulation and were immediately transferred to the laboratory. After quantifying the number of pods, the seeds were carefully removed and placed in properly identified plastic bags. Likewise, the leaves of the bean plants were collected by stratified sampling and were immediately taken to the Molecular and Cellular Biology Laboratory of the Faculty of Natural Sciences for analysis.

The biological material was placed in a forced air circulation oven at 40 °C (Shell ab Fx 1375, Swedesboro, NJ, USA) until reaching constant weight. Subsequently, the samples were ground in a mill (Thomas Wiley, Model 4 Scientific, Cornellus, OR, USA) with a sieve diameter of 0.55 mm. The powder samples were then stored in an ultra-freezer (REVCO last II, Asheville, NC, USA) at −80 °C until analysis.

### 4.4. Obtaining, Extracting and Characterizing Lectins and Protease Inhibitors from Tepary Bean Seeds

The Tepary bean seeds used were purchased in a local market (Arizpe community) at Hermosillo, Sonora, Mexico, and a sample was identified and deposited in the Dr. Jerzy Rzedowski Herbarium at the Faculty of Natural Sciences, Autonomous University of Queretaro, Mexico (ID: QMEX00007888). These seeds were planted and cultivated, and the resulting plants subjected to the treatments, after which the new seeds were harvested.

#### 4.4.1. Lectin Extraction and Purification

Lectins from Tepary bean seeds were extracted using approximately 100 g of defatted raw bean flour (DRBF) dissolved in 1 L deionized water following the methodology developed by our research group [4]. Briefly, a sequential precipitation with ammonium sulfate was performed from the crude protein extract (CPE), starting with 40% ammonium sulfate saturation. The protein precipitate was discarded (P40), and the supernatant was recovered and brought to 70% ammonium sulfate saturation to precipitate the proteins (P70). The precipitated proteins were collected after centrifugation at 39,200× *g* for 30 min. The pellet (P70) was dissolved in 15 mL of deionized water and dialyzed in a 3 kDa membrane (Spectrum Laboratory, Inc. Standard RC Tubing No. 9200676, Sigma Aldrich, St. Louis, MO, USA) against deionized distilled water at 4 °C, until it reached a 2 µΩ conductance. The resultant extract was named lectin inhibitor protein (LIP-70). This procedure was performed for each treatment (T1, T2 and T3). Subsequently, the physicochemical characterization of the LIP-70 extract was carried out as follows:

#### 4.4.2. Electrophoretic Profile SDS-PAGE

The electrophoretic profiles of the fractions recovered were obtained by SDS-PAGE in 10% polyacrylamide gels by the Laemmli method [36]. The amount of total protein in the fraction was previously determined by the Bradford method [37].

#### 4.4.3. Identification of Glycoproteins

Glycoproteins present in the polyacrylamide gels were stained following the Periodic-Acid Schiff staining technique [38,39].

#### 4.4.4. Agglutination Activity

Determination of the binding activity was carried out as described by Jaffé [40]. Briefly, serial twofold dilutions were made in 96-well plates, and 100 μL of PBS was placed as a negative control. Then 100 μL of the LIP-70 fraction was added in triplicate at a concentration of 1 mg/mL (protein concentration was previously determined). Subsequently, 50 μL of PBS was added to the rest of the plate, and serial dilutions were made, starting in the first well and moving to the following ones. Next, 50 μL of 2% type A^+^ human erythrocytes, which had previously been fixed with glutaraldehyde, was added to each well. This was then incubated at 37 °C for 2 h. The plaque was viewed under an inverted microscope, and the binding activity was determined using the following formula:

SAA = specific agglutinating activity (U/mg) expressed in units per mg of initial protein, n = last dilution with appreciable agglutination under the microscope, while mg is the amount of protein present in the sample.

#### 4.4.5. Enzyme Activity

From the LIP-70 fraction, the inhibitory activity of the protease inhibitor (PI) against bovine trypsin was determined with the substrate N-benzoyl-arginine-ethyl-ester (BAEE), according to the method developed by Schwert and Takenaka [41] and by using the substrate N-benzoyl-arginine-p-nitroanillide (BApNA) in a 96-well microplate spectrophotometer [42]. Inhibitory activity against chymotrypsin was determined by taking a 5 μL sample of LIP-70, to which was added 95 μL of 0.1 M pH 8 Tris-HCl buffer, 10 μL of bovine chymotrypsin and 10 μL of Suc-AAPF-pNA substrate, according to Erlanger’s method [42].

#### 4.4.6. Inhibition Zymogram

The detection of proteins with protease inhibitor activity was carried out by digestion with bovine trypsin, which was carried out according to the protocols of Ohlsson et al. [43] and Vinokurov et al. [44]. A 10% gel electrophoresis was performed according to the Laemmli system [36]. The gel was subsequently washed with distilled water for 5 min. A dilution of bovine trypsin (1 mg of protein/mL of water) was prepared. This dilution was placed on the gel and allowed to incubate for 4 h at 37 °C; then the gel was washed with water and stained with Coumassie blue to observe the presence of trypsin inhibitors.

### 4.5. Extraction of Phenolic Compounds from Leaves

For the preparation of the extracts, 200 mg of powdered leaf sample was weighed, and 10 mL of distilled water was added; the mixture was then placed in an ultrasonicator (model BRANSON 5510, EU) for 30 min at 42 kHz ± 6 at room temperature and under conditions of minimum lighting. Subsequently, the extract was filtered through Whatman paper (0.20 mm). The resulting solution was stored in amber flasks at −20 °C until analysis.

### 4.6. Determination of Total Phenolic Compounds

Total phenolic compounds (TPC) in all leaf extracts were determined spectrophotometrically using the Folin–Ciocalteu method [45]. Briefly, 12.5 µL of each extract was taken in triplicate and mixed with 32 µL of Folin–Ciocalteu reagent (1N) and 156 µL of 20% NaCo_3_. The resulting mixture was incubated in the absence of light for 2 h at room temperature. Subsequently, the absorbance was read at a wavelength of 750 nm in a Spectramax 250 Microplate Reader (Molecular Devices, Marshall Scientifica, Sunnyvale, CA, USA). A calibration curve was prepared using gallic acid as a standard. TPC was reported as mg gallic acid equivalents per 100 g of dry matter (mg GAE/100 g DM).

### 4.7. Determination of Total Flavonoids

Total flavonoid content (TFC) in leaf extracts was determined by using the modified colorimetric method developed by Zhishen [46,47]. Briefly, 31.25 µL of each extract was mixed in triplicate with 156 µL distilled water and 9.4 µL 5% NaNO2 and was allowed to incubate for 6 min. Then 10% AlCl3 was added, and the mixture was allowed to incubate for 5 min. Subsequently, 63 µL of NaOH (1M) and 35 µL of distilled water were added. The reaction mixture was kept at room temperature in the absence of light for 30 min, and finally, the absorbance was read at a wavelength of 510 nm in a Spectramax 250 Microplate Reader (Molecular Devices, Marshall Scientifica, Sunnyvale, CA, USA). A calibration curve was prepared using the catechin equivalent as a standard. The results were expressed as mg of (+)-catechin equivalents per 100 g of dry matter (mg CE/100 g DM).

### 4.8. Determination of Condensed Tannins

The condensed tannin content in leaf extracts was determined using Deshpande’s modified vanillin hydrochloric acid test [48]. Briefly, 40 µL of each extract was mixed in triplicate with 200 µL of a solution of 1% vanillin and 8% HCl in methanol in a 1:1 ratio. The resulting sample was incubated at room temperature and in the absence of light for 20 min. Finally, the absorbance was read at a wavelength of 492 nm in a Spectramax 250 Microplate Reader (Molecular Devices, Marshall Scientifica, Sunnyvale, CA, USA). The results were expressed in mg of catechin equivalents per 100 g of dry matter (mg CE/100 g DM).

### 4.9. Determination of Anthocyanins

Anthocyanins in leaf extracts were measured according to the method developed by Abdel and Hucl [49]. Briefly, 500 mg of dry sample was mixed with 12 mL of acidified ethanol (1N). Subsequently, the pH was adjusted to 1 with HCl (4N). Finally, 240 µL of the resulting sample was taken and read at a wavelength of 535 nm. The results were expressed in mg of cyanidin 3-glucoside equivalents (C3GE) per 100 g of DM.

### 4.10. Antioxidant Capacity

Two techniques were used to evaluate the antioxidant capacity of the Tepary bean leaves. The first, a 1,1-diphenyl-2-picrylhydrazyl (DPPH) assay, was conducted as reported by Dewanto et al. [47]. Briefly, the DPPH radical was prepared by mixing 1.5 mg of DPPH in 25 mL of reagent grade methanol. Subsequently, 20 µL of each of the samples were mixed with 200 µL of DPPH, and the resulting reaction was incubated for 1 h at room temperature and in the absence of light. Finally, the absorbance was read at a wavelength of 520 nm in a microplate reader. Antioxidant capacity was reported as μmol Trolox equivalents/100 g DM (μmol ET/g DM). Additionally, DPPH radical removal activity was calculated using different extract concentrations (1–10 mg/mL) and expressed as (%) inhibition. The other Trolox equivalent antioxidant capacity (TEAC) assay was carried out according to the method established by Re et al. [50]. Briefly, to generate the radical, 7 mM ABTS (2,2′-azinobis-3-ethylbenzothiazoline-6-6 sulfonic acid) was mixed with 2.45 mM potassium persulfate in water for 12 h at room temperature and in the absence of light. Then 20 µL of each of the samples were mixed with 230 µL of the ABTS dilution, and the resulting reaction was incubated for 1 h at room temperature and in the absence of light. Finally, the absorbance was read at a wavelength of 743 nm in a microplate reader.

The antioxidant capacity was expressed as (mmol TE/g DM). Furthermore, (%) inhibition was determined.

### 4.11. Statistical Analysis

Descriptive statistics were used as means and standard deviations to present the values of the analyzed variables. To compare the values of the variables studied, an analysis of variance (ANOVA) was used followed by a post hoc Tukey test to evaluate possible differences between the T1, T2 and T3 treatments. A 95% confidence interval and a significance level of *p* < 0.05 were used. A Pearson correlation analysis was performed between the components present in the Tepary bean leaves (total phenolic compounds, total flavonoids, condensed tannins and anthocyanins), including the antioxidant capacity analyzed by DPPH and by TEAC. The data were analyzed using R software (The R Foundation for Statistical Computing, Vienna, Austria), version 3.5.3 [51].

## 5. Conclusions

The stress induced by mechanical foliar damage after pod-filling in the Tepary bean plant (*Phaseolus acutifolius*) increased lectin binding units fivefold compared to the control group but did not affect enzymatic activity against trypsin (protease inhibitors). Likewise, mechanical foliar damage decreased seed production and did not significantly increase the levels of phenolic compounds in the leaf. The results show that controlled mechanical foliar damage could be a way to induce the overexpression of proteins of interest such as lectins present in the seed of Tepary beans. However, in terms of yield, it does not seem to be promising. For the production of specific bioactive molecules such as lectins from Tepary beans, the production of recombinant proteins through biotechnological methods appears to be more promising.

## Figures and Tables

**Table 1:** Number of leaves treated with mechanical damage and number of fresh and dry pods obtained from Tepary bean (*Phaseolus acutifolius*).

Treatment (Mechanical Damage)	Total Leaves/Plant	Treated Leaves/Plant	Fresh Pods/Plant	Dried Pods/Plant
T1 (0%)	58.8 ± 38.5 ^a^	0	11.2 ± 10.01 ^a^	29.2 ± 27.4 ^a^
T2 (50%)	60.4 ± 27.4 ^a^	30.2 ± 13.7	8.5 ± 8.3 ^ab^	28.9 ± 17.4 ^a^
T3 (80%)	57.9 ± 32.5 ^a^	46.3 ± 26.02	6.0 ± 96.9 ^b^	27.1 ± 20.7 ^a^

Number of plants per treatment (n = 120). Different letters in the same column indicate significant difference (*p* < 0.05). ANOVA analysis followed by a post hoc Tukey test.

**Table 2:** Yield of total pods and seeds of Tepary bean (*Phaseolus acutifolius*) by treatment.

Treatment (Mechanical Damage)	Total Fresh Pods	Diff.	Total Dry Pods	Diff.	Fresh and Dried Pods	Diff.	Seeds (g)	Diff. Quantity (%)
T1 (0%)	1285	0 (100%)	3468	0 (100%)	4753	0 (100%)	1890	0 (100%)
T2 (50%)	1015	−270 (78.9%)	3463	−5 (99.9%)	4478	−275 (94.2%)	1658	−232 (87.7%)
T3 (80%)	717	−568 (55.8%)	3250	−218 (93.7%)	3967	−786 (83.5%)	1615	−275 (85.4%)

The yield corresponds to 120 plants per treatment. The difference (Diff.) is the comparison of the resulting amount in the treatment taking as reference T1 (0% mechanical damage: control group).

**Table 3:** Purification of protein, lectin binding units and units of enzymatic activity against trypsin in the different treatments of Tepary bean plants (*Phaseolus acutifolius*).

Treatment (Mechanical Damage)	mg CPE/100 g DRBF	mg P40/100 g DRBF	mg P70/100 g DRBF	LBUs from LIP-70/mg Protein	UEA against Trypsin from LIP-70/mg Protein
T1 (0%)	990 ± 23 ^a^	640 ± 32 ^a^	490 ± 10 ^a^	1280 ± 760 ^a^	60.8 ± 26.9 ^a^
T2 (50%)	850 ± 19 ^a^	655 ± 28 ^a^	455 ± 40 ^a^	2560 ± 1108 ^b^	60.6 ± 18.3 ^a^
T3 (80%)	905 ± 18 ^a^	700 ± 28 ^a^	440 ± 32 ^a^	6542 ± 2893 ^c^	51.0 ± 19.3 ^a^

CPE: crude protein extract; DRBF: defatted raw bean flour; P40: discarded protein extract obtained at 40% saturation with ammonium sulfate; P70: protein extract obtained at 70% saturation with ammonium sulfate; LBUs: lectin binding units; LIP-70: lectin inhibitor protein P70; UEA: units of enzyme activity. Results are shown as the mean and a standard deviation of n = 9 determinations. Different letters indicate significant difference (*p* < 0.05) in the same column, using an ANOVA test followed by a post hoc Tukey test.

**Table 4:** Total phenolic compounds, total flavonoids and condensed tannins in Tepary bean (*Phaseolus acutifolius*) leaf.

Treatment (Mechanical Damage)	Total Phenols (mg GAE/100 g DM)	Total Flavonoids (mg CE/100 g DM)	Condensed Tannins (mg CE/100 g DM)	Total Anthocyanins (mg C3GE/g DM)
T1 (0%)	1374.45 ± 55.83 ^a^	797.27 ± 25.91 ^a^	1.51 ± 0.06 ^a^	0.79 ± 0.03 ^a^
T2 (50%)	1103.88 ± 38.66 ^b^	574.20 ± 29.19 ^b^	1.49 ± 0.09 ^a^	0.81 ± 0.02 ^a^
T3 (80%)	1285.13 ± 43.56 ^c^	767.15 ± 34.31 ^a^	1.48 ± 0.10 ^a^	0.73 ± 0.02 ^b^

GAE: gallic acid equivalents; CE: catechin equivalents; C3GE: cyanidin 3-glucosede equivalents; DM: dry matter. Results are shown as the average of n = 3 determinations ± one standard deviation. Different letters mean significant difference (ANOVA, post hoc Tukey test, *p* < 0.05) between extracts in the same analysis technique.

**Table 5:** Antioxidant capacity of the Tepary bean leaf (*Phaseolus acutifolius*) by the DPPH and ABTS methods.

Treatment (Mechanical Damage)	DPPH		ABTS	
	µmol TE/g DM	% of Inhibition	µmol TE/g DM	% of Inhibition
T1 (0%)	249.79 ± 10.30 ^a^	31.64 ± 4.46 ^a^	334.56 ± 13.62 ^a^	59.03 ± 5.07 ^a^
T2 (50%)	240.49 ± 4.58 ^b^	27.76 ± 1.92 ^b^	303.29 ± 13.61 ^b^	47.53 ± 4.97 ^b^
T3 (80%)	263.76 ± 5.83 ^c^	37.78 ± 2.57 ^c^	318.31 ± 6.70 ^c^	53.04 ± 2.52 ^c^

µmol ET: micromoles of Trolox equivalents; DM: dry matter. Results are shown as the average of n = 3 determinations ± one standard deviation. Different letters mean significant difference (ANOVA, post hoc Tukey test, *p* < 0.05) between extracts in the same antioxidant capacity technique.

## Data Availability

Not applicable.
